# Therapeutic strategy for acute appendicitis based on laparoscopic surgery

**DOI:** 10.1186/s12893-023-02070-y

**Published:** 2023-06-13

**Authors:** Masahiro Shiihara, Yasuhiro Sudo, Norimasa Matsushita, Takeshi Kubota, Yasuhiro Hibi, Harushi Osugi, Tatsuo Inoue

**Affiliations:** Department of Surgery, Kamifukuoka General Hospital, 931 Fukuoka Fujimino-Shi, Saitama, 356-0011 Japan

**Keywords:** Appendicitis, Laparoscopy, Complicated appendicitis, Uncomplicated appendicitis

## Abstract

**Purpose:**

The treatment strategies for acute appendicitis differ depending on the facility, and various studies have investigated the usefulness of conservative treatment with antibiotics, laparoscopic surgery, and interval appendectomy (IA). However, although laparoscopic surgery is widely used, the clinical strategy for acute appendicitis, especially complicated cases, remains controversial. We assessed a laparoscopic surgery-based treatment strategy for all patients diagnosed with appendicitis, including those with complicated appendicitis (CA).

**Methods:**

We retrospectively analysed patients with acute appendicitis treated in our institution between January 2013 and December 2021. Patients were classified into uncomplicated appendicitis (UA) and CA groups based on computed tomography (CT) findings on the first visit, and the treatment course was subsequently compared.

**Results:**

Of 305 participants, 218 were diagnosed with UA and 87 with CA, with surgery performed in 159 cases. Laparoscopic surgery was attempted in 153 cases and had a completion rate of 94.8% (145/153). All open laparotomy transition cases (*n* = 8) were emergency CA surgery cases. No significant differences were found in the incidence of postoperative complications in successful emergency laparoscopic surgeries. In univariate and multivariate analyses for the conversion to open laparotomy in CA, only the number of days from onset to surgery ≥ 6 days was an independent risk factor (odds ratio: 11.80; *P* < 0.01).

**Conclusion:**

Laparoscopic surgery is preferred in all appendicitis cases, including CA. Since laparoscopic surgery is difficult for CA when several days from the onset have passed, it is necessary that surgeons make an early decision on whether to operate.

**Supplementary Information:**

The online version contains supplementary material available at 10.1186/s12893-023-02070-y.

## Introduction

Acute appendicitis is one of the most common surgical conditions. However, the clinical presentation of appendicitis varies depending on the degree of inflammation and the patient’s background [1–3]. Recently, many reports have classified acute appendicitis as uncomplicated appendicitis (UA) and complicated appendicitis (CA). (1) CA is often defined as a gangrenous appendix, perforated appendix, or peri-appendiceal abscess [2, 3]. Traditionally, emergency surgery has been the gold standard for the treatment of acute appendicitis. However, because of the widespread use of laparoscopic surgery or conservative treatment options with antibiotics, treatment strategies have been diversifying and differ depending on the institution. Several studies have reported that conservative treatment with antibiotics may successfully treat most UA cases [4–6]. Furthermore, recent research has demonstrated that CA could also be treated with antibiotics [7, 8]. The efficacy of laparoscopic surgery or interval appendectomy (IA) after conservative treatment has also been proved in the past decades [8, 9]. Although consensus has not been reached, several reports have verified the usefulness of ​​laparoscopic surgery for CA cases. Therefore, we assessed a laparoscopic surgery-based treatment strategy for all patients diagnosed with appendicitis, including those with CA.

## Material and methods

### Study population

We retrospectively evaluated medical records of all patients diagnosed with acute appendicitis in our institution between January 2013 and December 2021. We excluded patients who did not have sufficient data.

### Diagnosis

Diagnosis of appendicitis is comprehensively determined by clinical findings, blood sampling data, and imaging (computed tomography or ultrasonography). Further, several experienced surgeons re-evaluated the diagnosis.

### Therapeutic strategy

Patients with suspected having with appendicitis initially underwent blood test and computed tomography (CT) (and ultrasonography). Antibiotics was administered as soon as the diagnosis was made. Emergency surgery was indicated for patients with peritoneal irritation sign, severe tenderness or sepsis at the first visit. We comprehensively consider not only patients’ imaging but also the symptoms and general conditions to determine whether or not emergency surgery is needed. Further, we performed emergency surgery for patients who deteriorated on study within 24 h after admission. Patients with mild symptoms were treated conservatively with fasting and antibiotics. Furthermore, they were recommended to undergo appendectomy on standby as an IA after more than a month.

### Surgical procedures

Laparoscopic appendectomy was typically performed in patients who were eligible for surgery, while open laparotomy was carried out in cases with possible tumours or extensive abscesses. We performed laparoscopic surgery with three ports, with an additional port added depending on intraoperative findings. Furthermore, we occasionally performed single-incision laparoscopic surgery as a part of the IA procedure. Appendiceal mesentery was resected using ultrasonic coagulating sears, and appendix was ligated by Endloop (Ethicon, Johnson and Johnson, Arlington, TX, USA). For cases in which the amount of intraoperative bleeding was very small or uncountable, the amount of bleeding was set to 5 ml for the convenience of analysis.

### Computed tomography assessment

Abdominal computed tomography (CT) was performed at the time of appendicitis diagnosis, and the images were evaluated by three or more experienced gastrointestinal surgeons. In this study, the images were reassessed retrospectively and classified into two categories (uncomplicated and complicated appendicitis) according to the previous literature. The CA group included gangrenous and perforated appendicitis. CT findings suggestive of CA included extraluminal appendicoliths, abscesses, appendiceal wall enhancement defects, and extraluminal defects [2, 3, 10].

### Histopathological findings

Pathological findings were evaluated by a board-certified pathologist and classified into four types based on the degree of inflammation: catarrhalis, phlegmonous, gangrenous, and chronic appendicitis.

### Statistical analysis

Continuous values are presented as medians. Between-group differences in qualitative and quantitative variables were analysed using two-tailed Fisher’s exact test, Pearson's chi-squared test, and Wilcoxon rank-sum test. Multiple logistic regression analysis of the factors contributing to the conversion of laparoscopic surgery to open laparotomy was performed. A *P* value of < 0.05 was considered statistically significant. Variables with a *P* value of < 0.05 according to a univariate analysis were included in the multivariate analysis. Each cut-off value was calculated using the Receiving Operating Characteristic curve. Propensity score matching was performed to adjust for patients’ background: age, sex, underlying diseases, and body mass index (BMI). All analyses were performed using JMP statistical software version 14.1.0 (SAS Institute Inc., Cary, NC, USA).

## Results

We assessed 308 patients who were diagnosed with acute appendicitis. Of these, 305 were analysed and three were ineligible because they had a final diagnosis of appendiceal mucinous adenoma or adenocarcinoma in the postoperative pathological findings. The median age of the patients was 37.4 years, and the number of male and female patients was 167 and 138, respectively. Surgery was performed in 159 patients, with laparoscopic surgery attempted on 153 patients, and an open laparotomy transition was required in eight of those cases. Thus, the completion rate of the laparoscopic surgery was 94.8% (145/153). Six of the 159 surgical cases indicated for open surgery because of extensive abscess formations, some were also suspected of tumours preoperatively. The baseline characteristics of the patients are presented in Supplementary Table 1 (Online Resource 1).

**Fig. 1:**
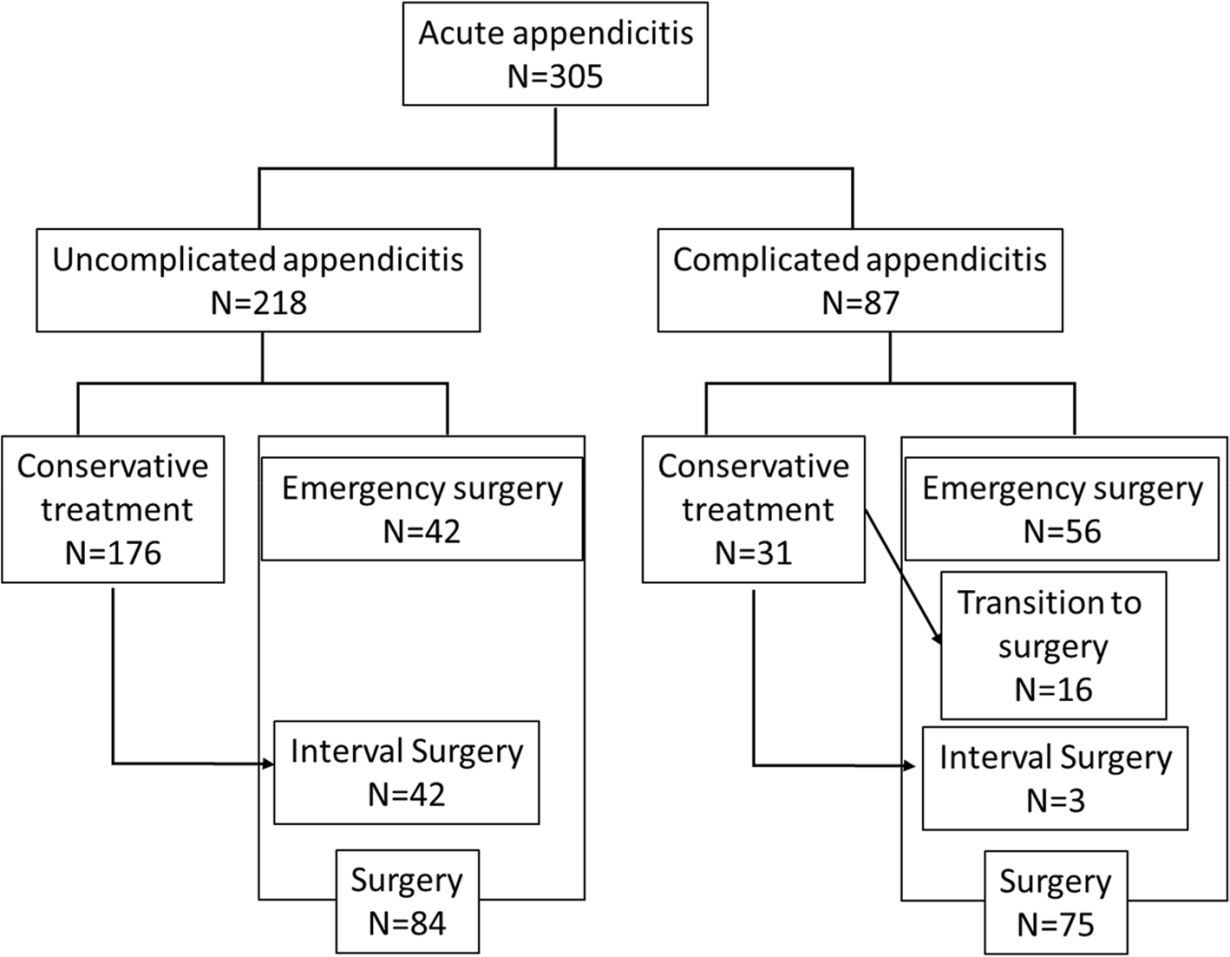
Classification of the 305 cases. Patients were divided into uncomplicated appendicitis (UA) and complicated appendicitis (CA) groups based on findings of computed tomography performed at the first visit

A total of 305 cases were classified into 218 UA and 87 CA cases based on CT findings at the time of the diagnosis (Table 1). Patients with CA were significantly older (*P* < 0.001) and had an increased likelihood of associated comorbidities (*P* = 0.01). In the preoperative laboratory data, the white blood cell count, neutrophil rate and count, and C-reactive protein level were significantly higher in the CA group. The UA cases included 38.5% (84/218) of patients who underwent surgery, and half of these (42/84) had an IA. All patients with UA (84/ 84) successfully underwent laparoscopic surgery. In the CA patients, surgery was performed in 86.2% (75/87) of cases. Of these, 16 cases were transitions after unsuccessful conservative treatment and three cases underwent IA. All patients who underwent IA (45/45) were successfully treated with laparoscopic surgery (Fig. 1).

**Table 1:** Comparison of the patients between UA and CA

Characteristics		UA group (*n* = 218)	CA group (*n* = 87)	*P* value
Background	Age, median (range), years	33.3 (8–91)	48.1 (4–88)	< 0.001
	Sex, Male/ Female, n	120/ 98	47/ 40	0.899
	Underlying diseases, n (%)	41 (18.8%)	33 (37.9%)	0.001
	BMI, median (range), kg/m^2^	21.3 (14.3–36.3)	21.7 (15.7–33.7)	0.828
Preoperative tatus	Initial symptoms, n (%) (Localized abdominal pain/ Extensive abdominal pain/ other)	205 (94%)/ 2 (0.9%)/ 11 (5.1%)	77 (88.5%)/ 7 (8.1%)/ 3 (3.5%)	0.004
	Body temperature, ℃	37.0 (35.8–39.8)	37.5 (36.0–39.8)	< 0.001
	Peritonitis on physical examination, n (%)	18 (8.3%)	43 (49.4%)	< 0.001
Preoperative data	Albumin, median (range), mg/dL	4.5 (3.1–5.6)	4.3 (2.8–5.7)	< 0.001
	CRP, median (range), mg/dL	1.45 (0.01–24.96)	8.65 (0.12–35.89)	< 0.001
	WBC, median (range), /μL	11,500 (3300–24,000)	12,900 (4200–27,600)	< 0.001
	Hb, median (range), mg/dL	14.2 (7.3–17.0)	13.7 (5.2–20.4)	0.253
	Neutrophils, median (range), /μL	9379 (1788–22,224)	10,919 (3360–23,543)	< 0.001
	Lymphocytes, median (range), /μL	13.9 (3.0–50.6)	9.2 (2.2–23.0)	< 0.001
	Platelet, median (range), 10^4^/μL	24.1 (8.0–44.4)	24. (11.0–47.4)	0.988
CT findings	Appendix diameter, median, (range), mm	10 (3–20)	13 (6.3–24)	< 0.001
	Fecalith, n	35 (17.4%)	44 (50.6%)	< 0.001
	Ascites, n	12 (5.5%)	31 (35.6%)	< 0.001
	Abscess, n	1 (0.5%)	42 (48.3%)	< 0.001
Initial treatment	Conservative/ Emergent surgery, n %	176 (80.7%)/ 42 (19.2%)	31 (35.6%)/ 56 (64.3%)	< 0.001
Results	Failure of conservative treatment, %	0.6% (1/ 176)	51.6% (16/ 31)	< 0.001
	Completion rate of laparoscopic surgery, %	100% (42/42)	87.5% (56/64)	0.021
	Interval appendectomy, %	23.9% (42/ 176)	9.7% (3/ 31)	0.098
Pathological findings	catarrhal/ phlegmonous/ gangrenous/ chronic	21 (27.3%)/ 25 (32.5%)/ 7 (9.1%)/ 24 (31.2%)	1 (2.3%)/ 18 (24.0%)/ 53 (70.7%)/ 3 (4.0%)	< 0.001

*BMI* Body mass index, *CA* Complicated appendicitis, *CRP* C-reactive protein, *CT* Computed tomography, *WBC* White blood cell, *UA* Uncomplicated appendicitis

In the comparison of emergency surgery groups between UA and CA, all patients in the UA group were treated completely with laparoscopic surgery, whereas in the CA group, 12.5% (8/64) ​​required open laparotomy (*P* = 0.021). In all eight cases, the cause of the transition to open laparotomy was the presence of severe adhesions. Table 2 shows the comparison of emergency surgery cases successfully treated laparoscopically between UA and CA groups. Significant differences were found in analysis of the operation duration (58.5 vs 86 min, *P* < 0.001), drain placement rate (33.3 vs 83.9%, *P* < 0.001), and postoperative days (5 vs 7 days, *P* = 0.04). There was no significant difference in the incidence of postoperative complications between the groups (*P* = 1.0). Similar results were obtained when propensity score matching was performed to adjust the patients’ background in Table 2 (Supplementary Table 2; Online Resource 1).

**Table 2:** Comparison of emergent surgery cases successfully treated by laparoscopy between UA and CA

Characteristics	UA emergency surgery *n* = 42	CA emergency surgery *n* = 56	*P* value
Operative time, median, (range), min	58.5 (31–113)	86 (42–278)	< 0.001
Blood loss, median, (range), ml	5 (5–5)	5 (5–200)	0.019
Drain placement	14 (33.3%)	47 (83.9%)	< 0.001
Postoperative complications, n (Clavien-Dindo grade II)	0 (0%)	1 (1.8%)	1.000
Postoperative hospital stay, median, (range), day	5 (3–38)	7 (4–24)	0.040
In-hospital mortality, n	0 (0%)	0(0%)	1.000

*CA* Complicated appendicitis, *UA* Uncomplicated appendicitis

In the comparison of the status between laparoscopically treated and open laparotomy converted cases in the CA group (Supplementary Table 3; Online Resource 1), the number of days from onset to surgery was significantly higher in the open transition group than in the laparoscopy completion group (2 vs 9 days, *P* < 0.001). CRP level at the first visit also tended to be higher in the open laparotomy transition group than in the laparoscopy completion group (7.0 vs 10.8, *P* = 0.054). Furthermore, the operative time, blood loss, postoperative complications, and postoperative hospital stay were all significantly higher in the open laparotomy converted group than in the laparoscopy completion group (Supplementary Table 2; Online Resource 1). In the univariate and multivariate analyses of the risk factors for the conversion to open laparotomy in CA, ≥ 6 days from onset to surgery was an independent risk factor (odds ratio 11.80; 95% confidence interval, 1.25–111.33; *P* < 0.01) (Table 3).

**Table 3:** Regression analysis of the factors for converting open laparotomy in CA

Factors			Patients	Converting open laparotomy	Univariable analysis		Multivariable analysis	
					Odds ratio (95% CI)	*P* value	Odds ratio (95% CI)	*P* value
Preoperative status	Age	≥ 54 year	20	5 (25.0%)	4.55 (0.97–21.44)	0.050		
	Sex	Male	37	7 (18.9%)	6.07 (0.70–52.61)	0.102		
	BMI	≥ 25 kg/m^2^	9	2 (22.2%)	2.19 (0.37–13.08)	0.389		
	Underlying diseases	with	22	2 (9.1%)	0.6 (0.11–3.26)	0.554		
	Past history of abdominal surgery	with	4	1 (25%)	2.52 (0.23–27.72)	0.449		
	Body temperature	≥ 37.5 ℃	33	4 (12.1%)	0.90 (0.20–3.95)	0.885		
	Peritonitis on physical examination, n (%)	with	37	2 (5.4%)	0.20 (0.04–1.08)	0.062		
	Days from onset to surgery	≥ 6 days	19	7 (36.8%)	25.67 (2.87–229.42)	0.004	11.80 (1.25–111.33)	0.010
preoperative data	Albumin	< 4.0 mg/dL	14	4 (28.6%)	4.6 (0.98–21.57)	0.053		
	CRP	≥ 4.5 mg/dL	40	8 (20.0%%)	N.A	0.004	N.A	0.191
	WBC	≥ 11,000 /μL	51	5 (9.8%)	0.36 (0.07–1.77)	0.226		
	Hb	≥ 15.9 mg/dL	12	4 (33.3%)	6 (1.24–28.99)	0.294		
	Neutrophils	≥ 9100 /μL	45	5 (11.1%)	0.40 (0.08–1.86)	0.246		
	Platelet	≥ 27.8 10^4^/μL	16	3 (18.8%)	1.98 (0.42–9.44)	0.401		
preoperative CT findings	Appendix diameter	≥ 14 mm	25	5 (20.0%)	2.83 (0.61–13.14)	0.18		
	Fecalith	with	39	2 (5.1%)	0.17 (0.03–0.93)	0.027	0.33 (0.05–2.18)	0.234
	Ascites	with	24	5 (20.8%)	3.25 (0.70–15.05)	0.125		
	Abscess	with	27	5 (18.5%)	2.58 (0.52–1.88)	0.216		
	ileus	with	28	3 (10.7%)	0.74 (0.16–34.19)	0.702		

*BMI* Body mass index, *CA* Complicated appendicitis, *CRP* C-reactive protein, *CT* Computed tomography, *WBC* White blood cell

## Discussion

In this study, we validated the usefulness of laparoscopic surgery for the treatment of appendicitis and attempted to evaluate the risk factors associated with converting to open laparotomy in CA. Because laparoscopic surgery could reduce postoperative complications and hospital stay, even in patients with CA, it is important to recognise these risk factors.

Treatment strategies for acute appendicitis vary depending on the facility and no definitive consensus exists, especially for CA cases. Some reports suggest that IA after conservative treatment is reasonable for CA, as IA reportedly has fewer postoperative complications and shorter postoperative hospital stays than emergency appendectomy [7]. However, for IA cases, the disadvantages are longer hospital stays and higher medical expenses [8]. Some studies have suggested that IA is unnecessary after the initial antibiotic treatment for CA [11, 12]. This is supported by the relatively low rates (approximately 10%) of appendicitis recurrence after conservative management, as well as high complication rates of the IA procedure [11, 12].

In recent decades, the use of laparoscopic surgery for appendicitis has become widespread. Advantages of laparoscopic surgery include a reduction in overall post-operative morbidity and surgical site infection, shorter postoperative hospital stay, less postoperative pain, and earlier postoperative recovery [13, 14]. Laparoscopic appendectomy has proven to be a safe alternative to open appendectomy in UA [15, 16]. However, the feasibility of laparoscopic surgery for CA has remained controversial. As a result of a meta-analysis, some studies concluded that laparoscopic surgery for CA has reduced surgical site infection rates compared to open surgery, with no difference with regard to intra-abdominal abscess complication rates [14, 17, 18]. The overall conversion rate from laparoscopic appendectomy to open appendectomy was reported to be approximately 10% [19]. To the best of our knowledge, there are no studies that verify the nature of cases that would convert to open laparotomy. In our study, all cases of UA were resolved by laparoscopic surgery, but it was revealed that cases of CA that were treated a few days after the onset of symptoms were more likely to convert to open laparotomy. The number of days from onset to surgery of ≥ 6 days was an independent risk factor for the conversion. The cause of this transition was the existence of strong adhesions in all the cases. It is important for surgeons to confirm the onset time during the patient’s first visit. For patients with CA with good general condition for whom some days from the onset have passed, IA could be a feasible alternative to emergency surgery. Resent multicenter study suggested that in-hospital delay of surgery in patients with CA was associated with a higher risk of a postoperative complication [20]. It is important that surgeons recognize and decide to operate patients with CA early as we suggested.

Several reports have discussed methods of appendectomy in laparoscopic surgery [21–23]. Zorzetti N et al. suggested the routine use of endloop for appendectomy even in case with complicated appendicitis because of cost effectivity and lower complications [21, 22]. Bao W et al. reported that purse-string sutures effectively reduced the incidence of postoperative complications after a laparoscopic appendectomy for CA [23]. We usually use endloop, but sometimes use endstapler for cases with necrotic appendicitis. Since there are still few reports on the method of excision, this is a topic for future research.

A recent topic on CA is the necessity of intraoperative peritoneal lavage. Whether intraoperative peritoneal lavage is effective for preventing postoperative intraabdominal abscesses for patients with CA remains controversial. Some study resulted that irrigation of peritoneal cavity during laparoscopic appendectomy could decrease the incidence of postoperative intraabdominal abscesses in patients with CA [24, 25]. Further, these patients also had faster postoperative recovery and lower hospital charges [24]. However, other studies denied the superiority of peritoneal lavage [26, 27]. One of the major problems is that the methods of peritoneal lavage are not standardized. A larg-scale multicenter study is needed.

This study has several limitations. First, it was a retrospective analysis with a small total number of CA cases, and its statistical power was too low for meaningful conclusions to be drawn for the broader application of the findings. Second, over the study period of 8 years, changes in the surgical indications, equipment, techniques, and perioperative management may have caused variations in the results.

## Conclusion

In conclusion, laparoscopic surgery is useful in all appendicitis cases, including CA. It is important to promptly decide whether emergency surgery is required in complicated cases. Surgeons should consider open laparotomy or conservative treatment for CA that is evaluated some days from the first onset of symptoms.

### Supplementary Information

**Additional file 1:** **Supplementary Table 1.** Patients characteristics. **Supplementary Table 2.** Comparison of emergent surgery cases successfully treated by laparoscopy between UA and CA after propensity score matching. **Supplementary Table 3.** Comparison of laparoscopic surgery completion cases and open laparotomy transition cases in emergency CA surgery.

## Data Availability

All publicly available data generated or analyzed during this study are included in this article. Further enquiries can be directed to the corresponding author.

## Abbreviations

CA: Complicated appendicitis
CT: Computed tomography
IA: Interval appendectomy
UA: uncomplicated appendicitis
