# Correction to: Therapeutic strategy for acute appendicitis based on laparoscopic surgery

**DOI:** 10.1186/s12893-023-02120-5

**Published:** 2023-08-07

**Authors:** Masahiro Shiihara, Yasuhiro Sudo, Norimasa Matsushita, Takeshi Kubota, Yasuhiro Hibi, Harushi Osugi, Tatsuo Inoue

**Affiliations:** Department of Surgery, Kamifukuoka General Hospital, 931 Fukuoka Fujimino-Shi, Saitama, 356-0011 Japan

Correction to: BMC Surgery 10.1186/s12893-023-02070-y.

Following publication of the original article [1], in this article the text has been inserted to the acknowledgement section and the same will read as follows:

The authors thank Editage (www.editage.jp) for English language editing. The authors are grateful to JPSKAKENHI #22K16548.
