# Supplementary material for: Therapeutic strategy for acute appendicitis based on laparoscopic surgery
Source: BMC Surg. 2023 Jun 13;23:161. doi: 10.1186/s12893-023-02070-y (PMC10265908; doi:10.1186/s12893-023-02070-y)
Supplement: Supplementary file 1 — Additional file 1: Supplementary Table 1. Patients characteristics. Supplementary Table 2. Comparison of emergent surgery cases successfully treated by laparoscopy between UA and CA after propensity score matching. Supplementary Table 3. Comparison of laparoscopic surgery completion cases and open laparotomy transition cases in emergency CA surgery. [file 12893_2023_2070_MOESM1_ESM.docx]

Supplementary Table 1: Patients characteristics

| Characteristics |  | n=305 |
| --- | --- | --- |
| Age, median (range), years |  | 37.4 (4–91) |
| Sex, Male/Female, n |  | 167/138 |
| BMI, median (range), kg/m^2^ |  | 21.4 (14.4–36.3) |
| Albumin, median (range), mg/dL |  | 4.4 (2.8–5.7) |
| CRP, median (range), mg/dL |  | 2.47 (0.01–35.89) |
| WBC, median (range), /μL |  | 12000 (3300–27600) |
| Hb, median (range), mg/dL |  | 14.1 (5.2–20.4) |
| Neutrophils, median (range), % |  | 81.8 (40.9–95.3) |
| Neutrophils, median (range), /μL |  | 9869 (1788–23543) |
| Lymphocytes, median (range), /μL |  | 11.5 (2.2–50.6) |
| Platelet, median (range), 10^4^/μL |  | 24.2 (8.0–47.4) |
| Appendix diameter, median, (range), mm |  | 11 (3–24) |
| Fecalith, n |  | 82 (26.9%) |
| Ascites, n |  | 43 (14.1%) |
| Abscess, n |  | 43 (14.1%) |
| Operation, n |  | 159 (52.1%) |
| Initial procedure (surgical cases) | Laparoscopic surgery / Open laparotomy | 151/ 8 |
|  | Completion rate of laparoscopic surgery | 94.7% (143/151) |
|  | Appendectomy/  Cecum partial resection/  Ileocecal resection | 153/ 3/ 3 |
| Postoperative complications, n (Clavien-Dindo grade ≥Ⅱ) |  | 6 |
|  | surgical site infection, n | 4 |
|  | ileus | 1 |
|  | colitis | 1 |
| In-hospital mortality, n |  | 0 |

BMI: Body mass index

Supplementary Table 2: Comparison of emergent surgery cases successfully treated by laparoscopy between UA and CA after propensity score matching

| Characteristics | UA emergency surgery n=34 | CA emergency surgery n=34 | *P* value |
| --- | --- | --- | --- |
| Operative time, median, (range), min | 58.5 (31–113) | 85.5 (42–167) | <0.001 |
| Blood loss, median, (range), ml | 5 (5–5) | 5 (5–200) | 0.039 |
| Drain placement | 14 (33.3%) | 30 (88.2%) | <0.001 |
| Postoperative complications, n (Clavien-Dindo grade II) | 0 (0%) | 1 (2.9%) | 1 |
| Postoperative hospital stay, median, (range), day | 5 (3–9) | 7 (4–24) | 0.001 |
| In-hospital mortality, n | 0 (0%) | 0 (0%) | 1 |

Supplementary Table 3: Comparison of laparoscopic surgery completion cases and open laparotomy transition cases in emergency CA surgery

| Characteristics | | Laparoscopic surgery n=56 | Converting open laparotomy n=8 | *P* value |
| --- | --- | --- | --- | --- |
| Preoperative status | Age, median (range), years | 44.1 (12–82) | 54.9 (21–73) | 0.286 |
|  | Sex, Male/Female, n | 30/ 26 | 7/ 1 | 0.124 |
|  | BMI, median (range), kg/m^2^ | 21.7 (15.7–33.7) | 22.5 (17.7–25.3) | 0.919 |
|  | Underlying diseases, n (%) | 23 (41.1%) | 2 (25%) | 0.704 |
|  | Past history of abdominal surgery, n (%) | 3 (5.4%) | 1 (12.5%) | 0.422 |
|  | Initial symptoms, n  (Localized abdominal pain/  Extensive abdominal pain/ other) | 52/ 3/ 1 | 6/ 1/ 1 | 0.184 |
|  | Body temperature (℃) | 37.6 (36.4–39.8) | 37.4 (36.7–38.8) | 0.931 |
|  | Peritonitis on physical examination, n (%) | 35 (62.5%) | 2 (25%) | 0.061 |
|  | Days from onset to surgery | 2 (0–36) | 9 (5–43) | <0.001 |
|  | Albumin, median (range), mg/dL | 4.4 (2.9–5.7) | 4.0 (3.0–4.9) | 0.105 |
|  | CRP, median (range), mg/dL | 7.0 (0.12–35.89) | 10.84 (4.51–29.14) | 0.054 |
|  | WBC, median (range), /μL | 13750 (8200–27600) | 12700 (9200–20200) | 0.479 |
|  | Hb, median (range), mg/dL | 14.1 (5.2–20.4) | 15.5 (11.6–16.1) | 0.440 |
|  | Neutrophils, median (range), /μL | 11721 (6265–23543) | 10699 (6918–19028) | 0.544 |
|  | Platelet, median (range), 10^4^/μL | 24.6 (14.8–47.4) | 26.4 (11.1–40.1) | 0.448 |
| Preoperative CT findings | Appendix diameter, median, (range), mm | 13 (6–20) | 14.5 (8–16) | 0.731 |
|  | Fecalith, n (%) | 37 (66.1%) | 2 (25%) | 0.048 |
|  | Ascites, n (%) | 19 (33.9%) | 5 (62.5%) | 0.139 |
|  | Abscess, n (%) | 22 (39.2%) | 6 (62.5%) | 0.266 |
|  | ileus | 25 (44.6%) | 3 (37.5%) | 1.000 |
| Operative status | Operative time, median, (range), min | 86 (42–278) | 117 (75–24) | 0.022 |
|  | Blood loss, median, (range), ml | 5 (5–200) | 75 (5–810) | <0.001 |
| Postoperative status | Postoperative complications, n (Clavien-Dindo grade ≥Ⅱ) | 1 (1.8%) | 2 (25.0%) | 0.039 |
|  | Postoperative hospital stay, median, (range), day | 7 (4–24) | 11.5 (11–51) | <0.001 |

BMI: Body mass index; CA: Complicated appendicitis; CRP: C-reactive protein; CT: Computed tomography; WBC: White blood cell.
